# The impact of same‐day and rapid ART initiation under the Universal Health Coverage programme on HIV outcomes in Thailand: a retrospective real‐life cohort study

**DOI:** 10.1002/jia2.26406

**Published:** 2025-01-02

**Authors:** Sirinya Teeraananchai, David C. Boettiger, Cheewanan Lertpiriyasuwat, Rattaphon Triamwichanon, Patchara Benjarattanaporn, Nittaya Phanuphak

**Affiliations:** ^1^ Department of Statistics Faculty of Science Kasetsart University Bangkok Thailand; ^2^ Biomedical Data Science Program Faculty of Science Kasetsart University Bangkok Thailand; ^3^ Kirby Institute University of New South Wales Sydney New South Wales Australia; ^4^ Division of AIDS and STIs Department of Disease Control Ministry of Public Health Nonthaburi Thailand Muang Thailand; ^5^ National Health Security Office Bangkok Thailand; ^6^ Joint United Nations Programme on HIV/AIDS Bangkok Thailand; ^7^ Institute of HIV Research and Innovation Bangkok Thailand

**Keywords:** HIV, antiretroviral therapy, same‐day ART, rapid ART, virological failure, linkage to care

## Abstract

**Introduction:**

Antiretroviral therapy (ART) initiation, regardless of CD4 count, has been recommended in Thailand since 2014, with same‐day initiation recommended since 2021. We assessed HIV treatment outcomes among Thai people living with HIV (PLHIV) by the time from HIV diagnosis to ART initiation under the Universal Health Coverage (UHC) programme and identified factors associated with virological failure (VF).

**Methods:**

PLHIV aged ≥15 years initiating ART between 2014 and 2022 were included from the UHC database. We categorized participants into four groups using the duration from HIV diagnosis to ART initiation: (1) ≤ 7 days (same‐day ART); (2) 8 days to <1 month; (3) 1–3 months; and (4) >3 months. Viral load (VL) was measured 6 months after starting ART, and annually thereafter. VF was defined as VL ≥1000 copies/ml. Factors associated with VF were analysed using competing risk models considering death and loss to follow‐up (LTFU) as competing events.

**Results:**

Among 252,239 PLHIV who started ART, the median age at initiation was 34 years (interquartile range [IQR]: 26–43 years). The median (IQR) pre‐ART CD4 count was 233 (76–420) cells/mm^3^. ART initiation occurred within 7 days for 25% (17% on the same day, 8% in 2–7 days), 24% in 8 days to <1 month, 23% in 1–3 months and 28% in >3 months. ART initiation within 7 days increased from 20% (2014–2016) to 32% (2021–2022). VF occurred with a rate of 3.11 (95% CI 3.07–3.159) per 100 person‐years (PYs). PLHIV initiating ART 8 days to 1 month were at lower risk of VF (aSHR 0.52, 95% CI 0.50–0.54) when compared to ART initiation >3 months. ART initiation within 7 days resulted in the lowest mortality (6%: 1.28 [95% CI 1.24–1.32] per 100 PYs), but the highest rate of LTFU (12%: 2.69 [95% CI 2.63–2.75] per 100 PYs) when compared to other ART initiation groups.

**Conclusions:**

Although ART initiation within 7 days has increased in Thailand, the overall rate of early initiation remains low. ART initiation within 1 month significantly lowered the risk of VF. ART initiation within 7 days significantly reduced mortality. To further optimize health outcomes, innovative strategies are urgently needed to promote earlier ART initiation in Thailand.

## 1 INTRODUCTION

The WHO has recommended that antiretroviral therapy (ART) should be initiated in all people living with HIV (PLHIV), regardless of CD4 cell count (treat‐all) since 2015 [1]. Additionally, since 2017, the WHO has recommended rapid ART initiation, including same‐day ART, for people newly diagnosed with HIV [2]. Previous studies in Africa indicate that treat‐all policies significantly increase rates of same‐day ART and rapid ART initiation [3, 4, 5, 6]. In Rwanda, rapid ART initiation increased from 44.4% to 78.9% between 2008 and 2018 following the introduction of the treat‐all policy [3]. Studies in Africa also suggest that individuals who receive same‐day ART initiation have higher rates of ART uptake and achieve faster viral suppression compared to those who initiate treatment more slowly [7, 8, 9, 10]. Moreover, a cohort study from Taiwan found that the proportion of PLHIV rapidly initiating ART increased from 33.8% in 2014 to 68.3% in 2017, which was associated with higher retention in care at 12 months, shortened interval from diagnosis to HIV suppression and reduced HIV transmission [11].

In Thailand, ART initiation regardless of CD4 count has been recommended since 2014, with same‐day ART initiation starting in 2021. Previous studies from real‐world settings have demonstrated that the widespread implementation of ART has contributed to a steady decline in HIV‐related mortality over the past decade before the era of treat‐all in 2014 [12]. However, addressing late diagnosis and delayed ART initiation is essential to further improving treatment outcomes for Thai PLHIV [12, 13]. In the era of treat‐all, new HIV cases among adults have decreased, along with the higher proportion of new HIV cases among males, during the period of 2014–2023 [14]. A pilot implementation of same‐day ART initiation launched in 2017 in Bangkok, found a retention rate of 75.3% at 12 months with 53.4% received viral load (VL) testing; 49.3% of those tested were virally suppressed [15]. A recent study explored the effect of the treat‐all policy on rapid ART initiation in youth living with HIV in Thailand, finding that the proportion initiating ART within 1 month after registration increased from 27% to 52% following the guideline changes [16]. To the best of our knowledge, no observational studies have reported on treatment outcomes among PLHIV initiating same‐day or rapid ART in Asia. In Thailand, PLHIV have been offered free HIV care and treatment through the Universal Health Coverage (UHC) programme since 2008. We assessed HIV outcomes among Thai PLHIV based on the time from HIV diagnosis to starting ART and identified factors associated with virological failure (VF), loss to follow‐up (LTFU) and death after ART initiation through the UHC programme.

## 2 METHODS

### 2.1 Universal Health Coverage programme

The UHC programme is the primary health insurance programme in Thailand for providing treatment and care to PLHIV [12, 16]. Hospitals and clinical sites enter laboratory results, clinical information and ART dispensing records into the database system of the UHC programme at the time of participants’ registration and during all subsequent visits. This information is used to reimburse hospitals for laboratory tests and medications provided. CD4 tests are conducted every 6 months, and VL is measured 6 months after starting ART, and annually thereafter. ART regimens are provided in accordance with the National HIV Treatment Guidelines [17]. The database is also linked with the National Death Registry, with vital status updated daily. The database is managed by the National Health Security Office (NHSO).

### 2.2 Study population

This cohort study included PLHIV aged ≥15 years, who were diagnosed and treated between 1 January 2014 and 31 December 2022 with at least one follow‐up. PLHIV were included if they had started ART with at least three drugs, consisting of a non‐nucleoside reverse transcriptase inhibitor (NNRTI) or protease inhibitor (PI) or dolutegravir (DTG) along with two nucleoside reverse‐transcriptase inhibitors (NRTIs). We excluded individuals who had received HIV treatment prior to enrolment in the UHC programme, as no clinical information was available for these individuals at the time of ART initiation. This study was approved by the Institutional Review Board of Kasetsart University Research Ethics Committee (KUREC‐HSR65/023), Thailand. A waiver of consent was granted for this data analysis, and the HIV database was de‐identified by the NHSO prior to analysis.

### 2.3 Definitions and outcomes

We classified PLHIV into four groups based on duration from diagnosis to ART initiation: (1) ≤ 7 days for same‐day ART; (2) 8 days to <1 month; (3) 1–3 months; and (4) >3 months. We also classified the periods of starting ART into three periods as 2014–2016, 2017–2020 and 2021–2022 to assess the trend of accessing same‐day ART. VF was defined as a VL ≥1000 copies/ml. PLHIV who discontinued follow‐up without undergoing VL testing after initiating ART were classified as LTFU, while those not yet due for a VL test were assumed to have no VF. Viral suppression (VS) after ART initiation was defined as VL < 50 copies/ml and VL < 1000 copies/ml as undetectable following WHO guidelines [18]. Mortality was confirmed in the death registry. LTFU was defined as not attending clinic visits for > 12 months, irrespective of whether individuals later returned to the programme after ART initiation. Pre‐ART CD4 count was defined as the closest result within a 12‐month window before, or up to 1 month after the date of ART initiation. Baseline was defined as the first date of ART initiation. We defined the HIV stage at ART initiation as follows: (1) Asymptomatic HIV for WHO Stage N or A; (2) Symptomatic HIV for WHO Stage B; and (3) AIDS for WHO Stage C.

### 2.4 Statistical analysis

Baseline characteristics, including demographics, registration year, region, history of opportunistic infection and CD4 count level, were summarized using descriptive statistics by ART initiation group. Formal comparisons of categorical characteristics between ART groups were performed using Pearson's Chi‐square test and continuous characteristics were compared using a Kruskal–Wallis test. Mortality, LTFU and VF rates were calculated per 100 person‐years of follow‐up (PY). The competing risk methods of Fine and Gray [19] was used to calculate sub‐distribution hazard ratios (SHR), to assess associations between characteristics and VF, with LTFU and death considered as competing events. Cox regression analysis was used to explore factors associated with mortality and LTFU after ART initiation. Covariates assessed in competing risks models and Cox regression included age, sex, ART initiation group, first regimen, pre‐ART CD4, country region, year of starting ART and HIV stages. We performed sensitivity analysis among PLHIV having the ascertained date of HIV diagnosis to classify the ART initiation group for validation of finding.

Variables with *p* < 0.10 in univariable screening were adjusted for in multivariable models. Statistical significance was identified using a two‐sided *p* value less than 0.05. Statistical analysis was performed with SAS version 9.4 (SAS Institute Inc, Cary, NC) and with Stata version 18 (StataCorp, College Station, TX).

## 3 RESULTS

A total of 255,939 PLHIV started ART through the UHC from 2014 to 2022. We excluded 2185 (1%) PLHIV who initiated with other ART‐based regimens (only NRTIs, NNRTI+PI, etc.), and 1515 (1%) had experienced ART. This resulted in an analysis population of 252,239 PLHIV.

### 3.1 Population characteristics

Of 252,239 PLHIV were eligible for this study and their baseline characteristics are shown in Table 1. Eighty‐four percent of PLHIV had a confirmed HIV diagnosis date; for the remaining PLHIV, the date of registration was used as a surrogate for the HIV diagnosis date to calculate the time to ART initiation. PLHIV started within 7 days in 25% (17% same‐day ART, 8% within 2–7 days), 24% started ART within 8 days to <1 month, 23% started between 1–3 months and 28% started ART >3 months after HIV diagnosis. The median duration from diagnosis to ART initiation among all PLHIV was 32 (interquartile range, IQR 8–111) days. The percentage of PLHIV who initiated ART within 7 days of HIV diagnosis increased from 20% in 2014–2016 to 32% in 2021–2022 (divided by a total number of years), with a median initiation time of 0 (IQR 0–3) days. The proportions of those initiating ART between 1–3 months and >3 months after diagnosis decreased during the same periods (from 25% to 19%, and from 35% to 21%), respectively (divided by a total number of years). Most PLHIV in our cohort were male with a median age at ART initiation of 34 (IQR 26–43) years. Twenty‐three percent were from the Northeastern region and 19% were from Bangkok. The highest percentage of PLHIV who started ART within 7 days (27%) were in Bangkok. Most PLHIV (89%) initiated NNRTI‐based regimens, followed by DTG‐based ART (7%) and PI‐based ART (4%). DTG‐based ART only became available under the UHC in 2021. Most (57%) PLHIV were asymptomatic. Among those diagnosed with HIV, 66% had CD4 counts available with a median of 204 (IQR 57–395) cells/mm^3^. Ninety‐two percent had pre‐ART CD4 available with a median of 233 (IQR 77–420) cells/mm^3^. The median pre‐ART CD4 count in PLHIV in the less than 7 days group was the highest compared to the other groups (319 vs. 202 vs. 145 vs. 260 cells/mm^3^, *p* <0.001).

**Table 1:** Characteristics at ART initiation by timing of ART initiation groups

	≤7 days	8 days to 1 month	>1–3 months	>3 months	Total
***N* (%)**	62,484 (25)	60,818 (24)	58,740 (23)	70,197 (28)	252,239 (100)
**HIV‐positive confirmed, *N* (%)**	52,994 (85)	55,067 (91)	51,813 (88)	51,083 (73)	210,957 (84)
**Sex, *N* (%)**					
Male	42,477 (68)	42,117 (69)	41,143 (70)	46,127 (66)	171,864 (68)
Female	20,007 (32)	18,701 (31)	17,597 (30)	24,070 (34)	80,375 (32)
**Age (years)**					
**Median (IQR) age at ART initiation (years)**	32 (25–41)	33 (25–43)	35 (26–44)	35 (28–43)	34 (26–43)
15–24 years	15,102 (24)	13,650 (22)	11,089 (19)	10,381 (15)	50,222 (20)
25–34 years	21,014 (34)	18,613 (31)	17,572 (30)	23,330 (33)	80,529 (32)
35–49 years	20,043 (32)	20,864 (34)	22,383 (38)	28,347 (40)	91,637 (36)
≥ 50 years	6325 (10)	7691 (13)	7696 (13)	8139 (12)	29,851 (12)
**First regimen**					
NNRTI based	51,845 (83)	54,249 (89)	53,968 (92)	64,211 (91)	224,273 (89)
PI based	4057 (6)	1753 (3)	1846 (3)	2865 (4)	10,521 (4)
DTG based	6582 (11)	4816 (8)	2926 (5)	3121 (4)	17,445 (7)
**HIV stage**					
Asymptomatic HIV	36,901 (59)	35,088 (58)	30,435 (52)	40,965 (58)	143,389 (57)
Symptomatic HIV	8398 (13)	11,419 (19)	10,623 (18)	9772 (14)	40,212 (16)
AIDS	17,185 (28)	14,311 (24)	17,682 (30)	19,460 (28)	68,638 (27)
**Year of ART initiation**					
2014–2016	18,270 (29)	18,093 (30)	23,296 (40)	32,283 (46)	91,942 (36)
2017–2020	28,101 (45)	29,112 (48)	27,017 (46)	29,111 (41)	113,341 (45)
2021–2022	16,113 (26)	13,613 (22)	8427 (14)	8803 (13)	46,956 (19)
**Region**					
Bangkok	17,127 (27)	9271 (15)	8192 (14)	12,320 (18)	46,910 (19)
Central	11,020 (18)	10,256 (17)	10,063 (17)	12,183 (17)	43,522 (17)
Eastern	6660 (11)	6860 (11)	6664 (11)	7861 (11)	28,045 (11)
Northeastern	12,298 (20)	15,465 (25)	14,416 (25)	15,204 (22)	57,383 (23)
Northern	9252 (15)	10,254 (17)	9157 (16)	10,828 (15)	39,491 (16)
Southern	5013 (8)	6593 (11)	7150 (12)	8435 (12)	27,191 (11)
Western	1114 (2)	2119 (3)	3098 (5)	3366 (5)	9697 (4)
**CD4 at HIV diagnosis, *N* (%)**	44,016 (70)	47,517 (78)	43,973 (75)	30,231 (43)	165,737 (66)
**Median CD4 at HIV diagnosis**	251 (92–420)	191 (55–363)	127 (38–313)	296 (84–488)	204 (57–395)
**Pre‐ART CD4, *N* (%)**	56,277 (90)	57,172 (94)	55,630 (95)	62,372 (89)	231,451 (92)
**Median pre‐ART CD4**	319 (158–503)	202 (61–378)	145 (42–332)	260 (105–445)	233 (77–420)
<200	17,725 (28)	28,612 (47)	32,525 (55)	25,616 (36)	104,478 (41)
200 to <350	13,539 (22)	12,244 (20)	10,104 (17)	13,958 (20)	49,845 (20)
350 to <500	11,173 (18)	8410 (14)	6693 (11)	10,598 (15)	36,874 (15)
≥500	13,840 (22)	7906 (13)	6308 (11)	12,200 (17)	40,254 (16)
Unknown	6207 (10)	3646 (6)	3110 (5)	7825 (11)	20,788 (8)

*Note*: Presented as *n* (%) for categorical data and median (interquartile range) for continuous data. The comparisons were performed using Pearson's Chi‐square tests or Fisher's exact test, as appropriate, for categorical data, and Kruskal–Wallis tests for continuous data. All characteristics were statistically significant differences among ART initiation groups at 0.05.

Abbreviations: ART, antiretroviral therapy; DTG, dolutegravir; NNRTI, non‐nucleoside reverse transcriptase inhibitor; PI, protease inhibitor; VL, viral load.

### 3.2 Virological treatment outcomes

**Figure 1:**
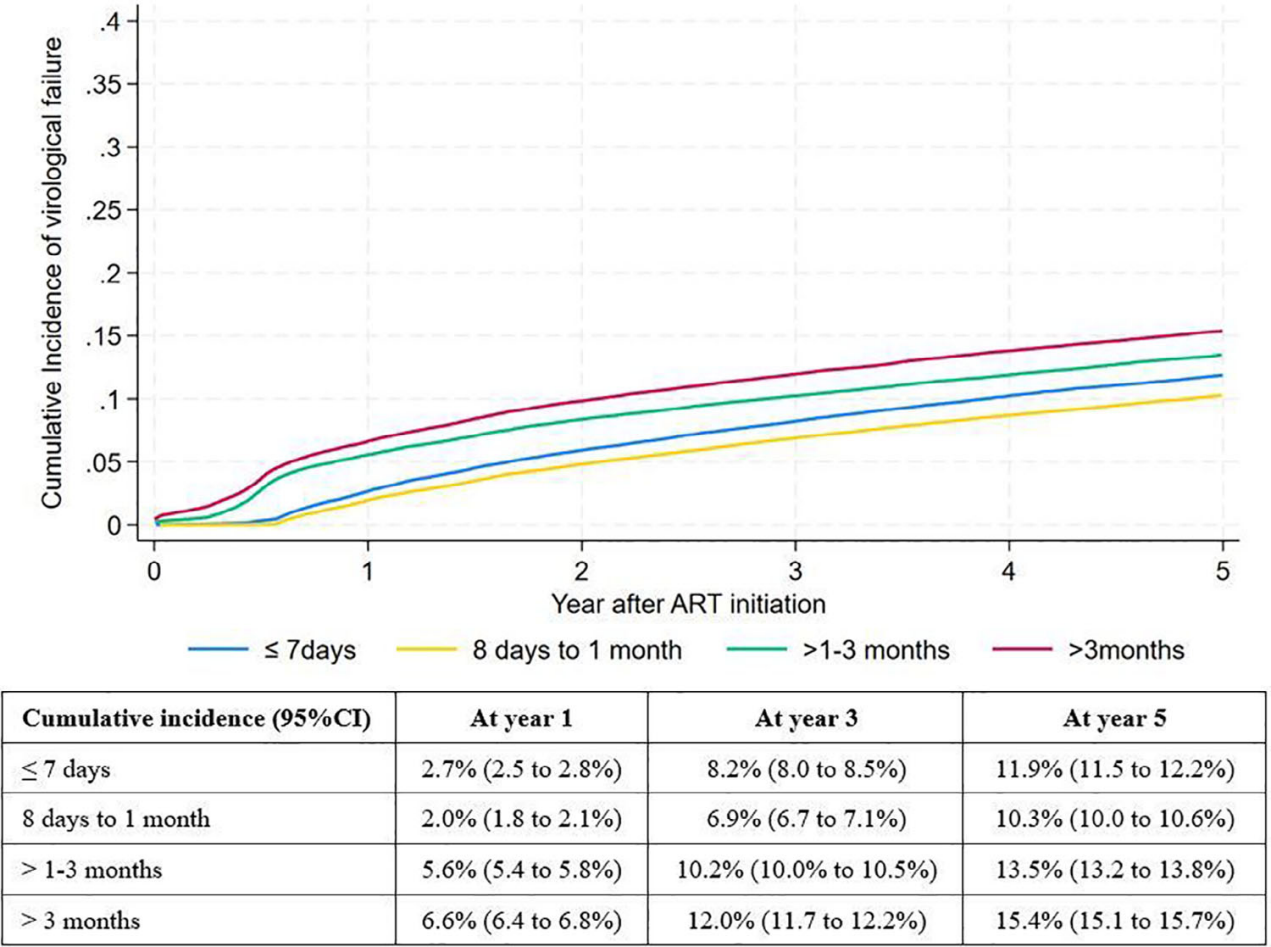
Cumulative incidence of virological failure by ART initiation group.

**Figure 2:**
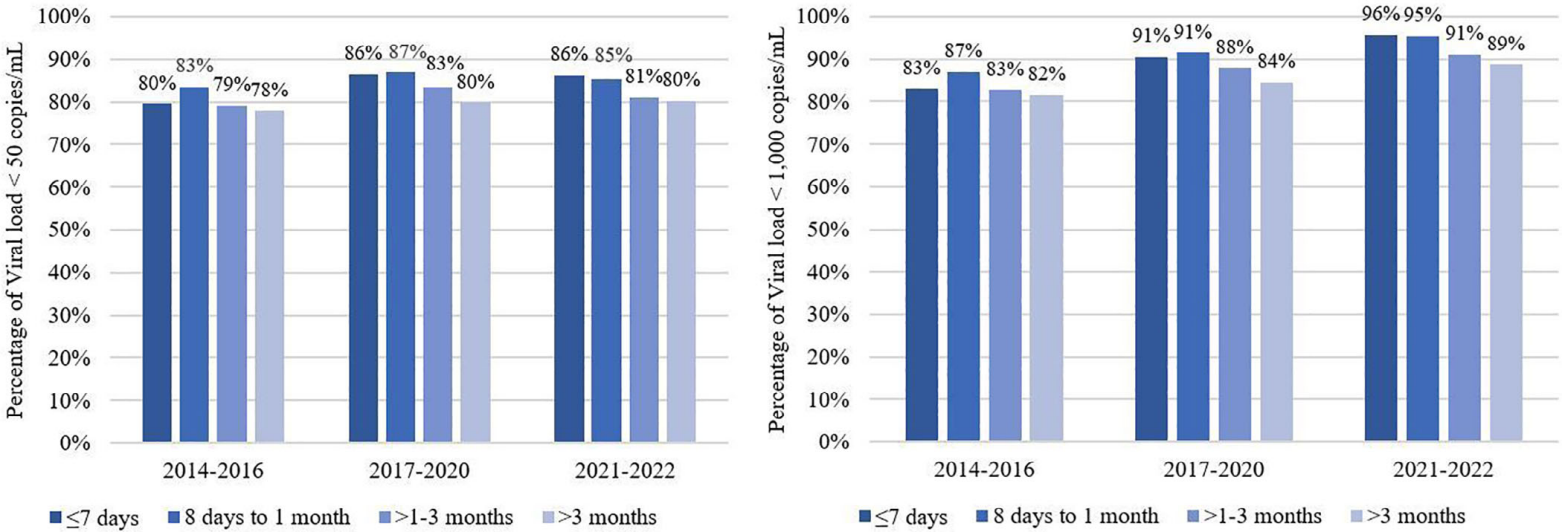
Proportion of virological suppression after ART initiation. *Note*: The denominator was the number of PLHIV having VL tests.

Of 252,239 PLHIV who started ART, 232,182 (92%) PLHIV had VL measurements after starting ART. The crude VF rate was 2.33 (95% CI 2.26–2.40) per 100 PYs for those who initiated ART from 8 days to <1 month, 2.76 (95% CI 2.69–2.83) per 100 PYs for those who started ART within 7 days, 3.35 (95% CI 3.27–3.43) per 100 PYs for those started ART between 1–3 months and 3.78 (95% CI 3.71–3.86) per 100 PYs for those initiating more than 3 months. The median duration from ART initiation to VF among PLHIV was 18 (IQR 8–38) months and was 13 (IQR 23–42) months after ART initiation in those initiating 8 days to less than 1 month group. The cumulative incidence of VF after ART initiation is shown in Figure 1. The proportion of VS among PLHIV starting ART on the same day or within 7 days and initiating ART 8 days to less than 1 month had an increasing trend from 2014–2016 to 2021–2022 which are shown in Figure 2.

### 3.3 Factors associated with virological failure

Table 2 shows factors associated with VF after ART initiation. In the adjusted multivariable model, VF was less likely in the group starting ART 8 days to less than 1 month (adjusted sub‐hazard rate, aSHR 0.52 [95% CI 0.50–0.54]), starting less than 7 days (aSHR 0.71 [95% CI 0.69–0.74]) and starting between 1–3 months (aSHR 0.74 [95% CI 0.72–0.76]) versus those starting more than 3 months after HIV diagnosis. Females had an 11% (aSHR 1.11 [95% CI 1.08–1.14]) higher risk of VF than males. PLHIV aged between 15 and 24 years (aSHR 2.76, 95% CI 2.62–2.91) were more likely to have VF compared to those aged ≥ 50 years at ART initiation. PLHIV with pre‐ART CD4 counts <200 cells/mm^3^ (aSHR 2.79, 95% CI 2.68–2.91), 200 to <350 cells/mm^3^ (aSHR 1.52, 95% CI 1.45–1.60) and 350 to <500 cells/mm^3^ (aSHR 1.16, 95% CI 1.10–1.22) had higher VF rates compared to those with baseline CD4 counts ≥500 cells/mm^3^. PLHIV who started ART with NNRTI‐ and PI‐based ART regimens had a higher risk of VF compared to those who started with DTG‐based ART regimens. PLHIV from the Northeastern regions (aSHR 0.91, 95% CI 0.88–0.95) were significantly less likely to have VF compared to those in Bangkok. The year of ART initiation and HIV stage were not associated with VF after adjusting for other factors.

**Table 2:** Factors associated with virological failure after ART initiation

	VF, *N* (%)	Univariable		Multivariable	
		SHR (95% CI)	*p*	aSHR (95% CI)	*p*
**ART initiation group**			<0.001		<0.001
≤7 days	5,426 (9)	0.71 (0.69–0.73)		0.71 (0.69–0.74)	
8 days to 1 month	4,518 (7)	0.59 (0.57–0.61)		0.52 (0.50–0.54)	
>1–3 months	6,913 (12)	0.87 (0.85–0.90)		0.74 (0.72–0.76)	
>3 months	9,749 (14)	reference (1)		reference (1)	
**Sex**			0.002		<0.001
Male	17,562 (10)	reference (1)		reference (1)	
Female	9,044 (11)	1.04 (1.02–1.07)		1.11 (1.08–1.14)	
**Age at ART initiation (years)**			<0.001		<0.001
15–24 years	6,565 (13)	2.12 (2.01–2.22)		2.76 (2.62–2.91)	
25–34 years	9,435 (12)	1.78 (1.70–1.87)		1.92 (1.83–2.02)	
35–49 years	8,654 (9)	1.36 (1.30–1.43)		1.33 (1.26–1.39)	
≥ 50 years	1,952 (7)	reference (1)		reference (1)	
**First regimen**			<0.001		<0.001
NNRTI‐based ART	24,295 (11)	2.60 (2.31–2.92)		2.29 (2.01–2.61)	
PI‐based ART	2,026 (19)	4.29 (3.79–4.86)		3.95 (3.44–4.54)	
DTG‐based ART	285 (2)	reference (1)		reference (1)	
**HIV stage**			<0.001		0.80
Asymptomatic HIV	13,644 (10)	ref		ref	
Symptomatic HIV	4,355 (11)	1.09 (1.06–1.13)		1.05 (1.02–1.09)	
AIDS	8,607 (13)	1.21 (1.18–1.24)		1.13 (1.09–1.16)	
**Year at ART initiation**			<0.001		0.05
2014–2016	14,184 (15)	ref		ref	
2017–2020	10,826 (10)	0.92 (0.89–0.94)		0.99 (0.96–1.01)	
2021–2022	1,596 (3)	0.71 (0.67–0.74)		0.94 (0.89–1.00)	
**Pre‐ART CD4, cells/mm^3^**			<0.001		<0.001
<200	14,852 (14)	2.21 (2.12–2.3)		2.79 (2.68–2.91)	
200 to <350	4,583 (9)	1.35 (1.29–1.42)		1.52 (1.45–1.60)	
350 to <500	2,744 (7)	1.10 (1.04–1.16)		1.16 (1.10–1.22)	
≥500	2,706 (7)	ref		ref	
Unknown	1,721 (8)	1.29 (1.21–1.37)		1.38 (1.30–1.46)	
**Region**			<0.001		0.04
Bangkok	4,600 (10)	ref		ref	
Central	4,809 (11)	1.12 (1.07–1.17)		1.04 (0.99–1.08)	
Eastern	3,155 (11)	1.17 (1.12–1.23)		1.07 (1.02–1.12)	
Northeastern	5,749 (10)	1.06 (1.02–1.10)		0.91 (0.88–0.95)	
Northern	4,133 (10)	1.05 (1.01–1.10)		0.97 (0.93–1.01)	
Southern	3,134 (12)	1.17 (1.11–1.22)		1.08 (1.03–1.13)	
Western	1,026 (11)	1.09 (1.02–1.17)		0.95 (0.88–1.01)	

Abbreviations: ART, antiretroviral therapy; aSHR, adjusted sub‐distribution hazard ratio; DTG, dolutegravir; NNRTI, non‐nucleoside reverse transcriptase inhibitor; PI, protease inhibitor; SHR, sub‐distribution hazard ratio; VF, virological failure; 95% CI, 95% confidence interval.

### 3.4 Attrition outcomes after ART initiation

The median duration on ART of PLHIV was 3.80 (IQR 1.53–6.25) years with a total of 1,244,409 PYs of follow‐up. We found that 10% of PLHIV (*n* = 25,845) died after ART initiation with a crude mortality of 2.11 (95% CI 2.08–2.13) per 100 PYs. The mortality rates of PLHIV decreased as the time to ART initiation shortened. The lowest mortality rate was in the less than 7 days group (1.28 [95% CI 1.24–1.32] per 100 PYs) followed by those in 8 days to less than 1 month (2.04 [95% CI 1.99–2.10] per 100 PYs) group. PLHIV who started ART between 1–3 months and >3 months after diagnosis had the highest mortality (2.49 [95% CI 2.44–2.55] per 100 PYs and 2.49 [95% CI 2.44–2.54] per 100 PYs), respectively. Moreover, 10% (*n* = 22,819) of PLHIV were LTFU including 997 (4%) who died after LTFU. The crude LTFU rate was (1.83 [95% CI 1.81–1.86] per 100 PYs). PLHIV started less than 7 days had the highest crude rate of LTFU (2.51 [95% CI 2.45–2.57] per 100 PYs), followed by those starting ART from 8 days to less than 1 month (2.27 [95% CI 2.21–2.32] per 100 PYs). The lowest LTFU rate was found in the group that initiated ART between 1–3 months (1.37 [95% CI: 1.32–1.41 per 100 PYs]) and more than 3 months after diagnosis (1.37 [95% CI: 1.34–1.41 per 100 PYs]).

### 3.5 Factors associated with mortality and lost to follow‐up after ART initiation

Factors associated with mortality after ART initiation are summarized in Table 3. In multivariate analysis, mortality was higher among PLHIV starting ART 8 days to <1 month (adjusted hazard ratio [aHR] 1.36; 95% CI 1.30–1.42), 1–3 months (aHR 1.58, 95% CI 1.51–1.64) and >3 months (aHR 1.74, 95% CI 1.68–1.81) compared to those starting ART less than 7 days after HIV diagnosis. Males had a 24% (95% CI 1.21–1.27) higher risk of mortality after ART initiation than females. We found that baseline age ≥ 50 years (aHR 3.09, 95% CI 2.94–3.24), age 35–49 years (aHR 1.94, 95% CI 1.86–2.03) and age 25–34 years (aHR 1.32, 95% CI 1.26–1.38) were associated with a higher risk of death compared to those aged 15–24 years. PLHIV with AIDS stage (aHR 1.46, 95% CI 1.42–1.50) and symptomatic HIV stage at baseline (aHR 1.34, 95% CI 1.29–1.38) had a higher risk of mortality than those who were asymptomatic. PLHIV with pre‐ART CD4 < 350 cells/mm^3^ and unknown CD4 had a higher risk of mortality compared with those who had baseline CD4 ≥ 350 cells/mm^3^. In an adjusted multivariate model for LTFU, PLHIV initiating ART from 8 days to 1 month had a 13% higher risk of being LTFU compared to those starting ART less than 7 days. Predictors of higher LTFU included being male, aged less than 25 years, starting with DTG‐based ART, having asymptomatic HIV and having pre‐ART CD4 ≥ 350 cells/mm^3^.

**Table 3:** Factors associated with mortality and lost to follow‐up

Covariates	Died, *N* (%)	Mortality				LTFU, *N* (%)	Lost to follow‐up			
		Univariable		Multivariable			Univariable		Multivariable	
		HR (95% CI)	*p*	aHR (95% CI)	*p*		HR (95% CI)	*P*	aHR (95% CI)	*p*
**ART initiation group**										
≤7 days	3,639 (6)	reference	<0.001	reference	<0.001	7196 (12)	reference	<0.001	reference	<0.001
8 days to 1 month	5,627 (9)	1.59 (1.53–1.66)		1.36 (1.30–1.42)		6341 (10)	1.62 (1.56–1.68)		1.13 (1.09–1.17)	
>1 to 3 months	7,384 (13)	2.04 (1.96–2.13)		1.58 (1.51–1.64)		4119 (7)	1.46 (1.41–1.51)		1.05 (1.01–1.09)	
>3 months	9,195 (13)	2.08 (2.00–2.16)		1.74 (1.68–1.81)		5163 (7)	0.96 (0.92–1.00)		1.01 (0.97–1.05)	
**Sex**										
Male	18,033 (10)	1.12 (1.09–1.15)	<0.001	1.24 (1.21–1.27)	<0.001	16,070 (9)	1.13 (1.10–1.16)	<0.001	1.01 (0.98–1.04)	0.37
Female	7,812 (10)	reference		reference		6749 (8)	reference		reference	
**Age at ART initiation (years)**										
15–24 years	2,532 (5)	reference	<0.001	reference	<0.001	6,154 (12)	reference	<0.001	reference	<0.001
25–34 years	6,357 (8)	1.52 (1.45–1.59)		1.32 (1.26–1.38)		7,317 (9)	0.73 (0.71–0.76)		0.76 (0.74–0.79)	
35–49 years	11,517 (13)	2.42 (2.32–2.52)		1.94 (1.86–2.03)		6,764 (7)	0.60 (0.58–0.62)		0.69 (0.66–0.71)	
≥50 years	5,439 (18)	3.79 (3.61–3.97)		3.09 (2.94–3.24)		2,584 (9)	0.73 (0.70–0.76)		0.68 (0.65–0.71)	
**First regimen**										
NNRTI based	24,373 (11)	1.84 (1.69–2.00)	<0.001	1.89 (1.72–2.07)	<0.001	11,693 (5)	0.04 (0.04–0.04)	<0.001	0.18 (0.17–0.18)	<0.001
PI based	926 (9)	1.38 (1.24–1.54)		1.91 (1.71–2.14)		712 (7)	0.05 (0.05–0.06)		0.29 (0.27–0.32)	
DTG based	546 (3)	reference		reference		10,414 (60)	reference		reference	
**HIV stage**										
Asymptomatic HIV	11,213 (8)	reference	<0.001	reference	<0.001	15,042 (10)	reference	<0.001	reference	<0.001
Symptomatic HIV	5,134 (13)	1.62 (1.56–1.67)		1.34 (1.29–1.38)		3,308 (8)	0.78 (0.75–0.81)		0.97 (0.94–1.01)	
AIDS	9,498 (14)	1.70 (1.65–1.74)		1.46 (1.42–1.50)		4,469 (7)	0.61 (0.59–0.63)		0.87 (0.84–0.90)	
**Year at ART initiation**										
2014–2016	12,994 (14)	reference	<0.001	reference	<0.001	2,210 (2)	reference	<0.001	reference	0.044
2017–2020	10,387 (9)	0.83 (0.81–0.85)		0.89 (0.86–0.91)		4,750 (4)	1.75 (1.66–1.85)		1.84 (1.75–1.94)	
2021–2022	2,464 (5)	0.76 (0.73–0.80)		0.94 (0.89–0.98)		15,859 (34)	14.08 (13.44–14.75)		12.01 (11.4–12.65)	
**Pre‐ART CD4, cells/mm^3^**										
<350	19,476 (13)	3.76 (3.61–3.91)	<0.001	2.96 (2.84–3.08)	<0.001	10,690 (7)	0.93 (0.90–0.96)	<0.001	0.96 (0.93–0.99)	<0.001
≥350	2,689 (3)	reference		reference		5,906 (8)	reference		reference	
Unknown	3,680 (18)	6.53 (6.21–6.86)		6.27 (5.97–6.59)		6,223 (30)	4.91 (4.74–5.09)		3.39 (3.26–3.51)	
**Region**										
Bangkok	3,203 (7)	reference	<0.001	reference	<0.001	3,972 (8)	reference	<0.001	reference	<0.001
Central	4,632 (11)	1.57 (1.50–1.64)		1.39 (1.33–1.46)		4,094 (9)	1.12 (1.07–1.17)		1.24 (1.18–1.29)	
Eastern	2,797 (10)	1.48 (1.41–1.56)		1.37 (1.30–1.44)		2,612 (9)	1.11 (1.06–1.17)		1.30 (1.24–1.37)	
Northeastern	6,400 (11)	1.69 (1.62–1.77)		1.56 (1.49–1.63)		5,267 (9)	1.11 (1.06–1.15)		1.20 (1.15–1.25)	
Northern	4,199 (11)	1.57 (1.50–1.64)		1.41 (1.35–1.48)		3,556 (9)	1.07 (1.02–1.12)		1.18 (1.13–1.24)	
Southern	3,479 (13)	1.91 (1.82–2.00)		1.62 (1.54–1.70)		2,395 (9)	1.05 (1.00–1.11)		1.28 (1.21–1.34)	
Western	1,135 (12)	1.77 (1.65–1.89)		1.48 (1.39–1.59)		923 (10)	1.14 (1.06–1.23)		1.31 (1.22–1.41)	

*Note*: *N* (%) was divided by a total of row.

Abbreviations: aHR, adjusted hazard ratio; ART, antiretroviral therapy; DTG, dolutegravir; HR, hazard ratio; LTFU, lost to follow‐up; NNRTI, non‐nucleoside reverse transcriptase inhibitor; PI, protease inhibitor; 95% CI, 95% confidence interval.

We performed sensitivity analysis among PLHIV who had the ascertained date of HIV diagnosis to classify the ART initiation group. The results are summarized in Tables S1–S3 and are consistent with our main findings.

## 4 DISCUSSION

This study analysed routine clinical data from the UHC programme in Thailand following guideline changes in 2014, which recommended initiating ART for all PLHIV regardless of CD4 count, and the introduction of same‐day ART initiation in 2021. Most PLHIV started ART rapidly, with a median time of ART initiation of 1 month and nearly one‐fourth initiated ART within a week after HIV diagnosis. The results indicate that starting ART within 1 month of HIV diagnosis was associated with lower rates of VF and mortality, but higher rates of LTFU. Same‐day or rapid ART initiation showed an increasing trend in VS during the study period. Our findings support the recent WHO recommendations and Thai National treatment guidelines, which advise that PLHIV should begin treatment soon after diagnosis to reduce mortality and improve treatment outcomes.

VL testing is the key monitoring to indicate HIV treatment outcomes and detecting treatment failure early [18, 20]. Our finding aligns with a related study in Thai youth living with HIV which reported that initiating ART within 1 month after diagnosis resulted in the lowest rate of VF, and that younger individuals were more likely to experience VF [21]. When controlling for other factors, the risk of VF was higher in those who started ART at a younger age, with a CD4 count < 200 cells/µl and those who delayed ART initiation for more than 3 months after diagnosis compared to those who started ART within 1 month. These results are consistent with other studies in Asia, where younger individuals with lower CD4 counts were more likely to experience treatment failure [20, 21]. Additionally, the previous study indicates that early ART initiation leads to durable VS [22] and same‐day ART and ART initiation within 1 month were equally likely to achieve VS. This is similar to our finding which indicated a high proportion of VS among PLHIV initiating ART on the same‐day or within 1 month.

Despite the reduction in mortality associated with starting ART on the same day, a substantially higher proportion of these groups were LTFU compared to those initiating ART after more than 1 month after diagnosis. This finding is consistent with cohort studies from Eswatini, Ethiopia, South Africa and Uganda [6, 23, 24, 25, 26]. Some evidence suggests that PLHIV who started ART on the same day as HIV diagnosis may receive improper counselling which could increase the risk of LTFU [25, 27]. Therefore, there is a need for enhanced counselling, adherence support and interventions to improve adherence, retention and monitoring during the early follow‐up period [25, 28, 29]. Thailand has streamlined HIV diagnosis, integrated HIV care into primary healthcare including community‐led clinics, issued 2021 National Service Manual for Promoting Same‐Day ART Initiation and held a series of training for healthcare workers to raise awareness and build capacity. Moving forward, efforts should focus on using the country's routine HIV quality assurance and quality improvement mechanisms to ensure the fidelity of same‐day ART initiation service components throughout all levels of healthcare facilities. To enhance health equity, Thailand must further simplify ART initiation through telehealth and task sharing with community health workers to allow ART initiation to happen at community‐led clinics where and when HIV diagnosis is made. Undetectable equals Untransmittable (U = U) communication must also be consistently integrated into the HIV care cascade as a main motivation for HIV testing, ART initiation and ART maintenance, as well as being used as a tool to address HIV‐related stigma and discrimination. Other countries can learn from Thailand's integration of HIV services into a universal healthcare system and the importance of addressing local barriers to implementing same‐day ART initiation.

A strength of this study is the description of HIV outcomes following the implementation of same‐day ART guidelines in Thai PLHIV who were followed‐up in a large real‐life setting cohort. This study also accurately determined the proportion of truly LTFU from care through linkage with the death registry, along with assessing outcomes in PLHIV based on the timing of ART initiation. However, this study has several limitations. First, we defined the date of registration in care as a surrogate for the date of HIV diagnosis. This might introduce bias by shortening the time from HIV diagnosis to ART initiation, as some individuals (16%) may have been diagnosed from HIV testing centres or private clinics and re‐tested at UHC facilities upon registration. In Thailand, HIV testing and ART initiation can happen in both public and private healthcare facilities at different levels from regional hospitals to provincial hospitals, district hospitals and primary health centres. There are differences in structural and cultural settings and resource availability across facilities which could affect the acceptability and feasibility of same‐day ART initiation. However, we did not have information to categorize different types of healthcare facilities in the database. However, in a sensitivity analysis excluding cases with unknown diagnosis dates, the main study findings remained unchanged. Second, approximately 3% of PLHIV did not undergo VL testing due to being LTFU, while those not yet due for a VL test were assumed to have no VF (7%). To minimize bias, we employed a competing risks model to account for LTFU and deaths, rather than excluding these cases, ensuring more accurate outcome estimates. No significant differences were observed by socio‐demographic characteristics in this study. Third, the database lacked complete information on the source of HIV exposure, so we could not analyse priority populations directly (e.g. men who have sex with men, injection drug use, etc.). The database did not record details on socio‐economic factors, such as gender identity, caregiver status, education status, occupation and income, limiting our ability to examine how these factors might influence both access to rapid ART and treatment outcomes. Last, this study was not able to assess important factors influencing the decision to start ART including symptoms and signs of meningitis, psychosocial readiness, and various forms of stigma and discrimination. We were also unable to explore site‐specific data related to counselling practices, staff attitudes or transfer‐out rates, which may have influenced the retention of early ART initiators. Future research should incorporate qualitative data or additional variables to explore reasons for LTFU, improving findings’ interpretation and informing better ART retention strategies.

## 5 CONCLUSIONS

In conclusion, ART initiation within 7 days became more common in Thailand over time although this still occurred in less than one‐third of PLHIV between 2021 and 2022. PLHIV with ART initiation within 1 month had a significantly lower rate of VF. Starting ART on the same‐day or within 7 days of HIV diagnosis significantly reduced mortality but was associated with a high rate of LTFU. Early ART initiation after HIV diagnosis has improved treatment outcomes and reduced mortality in Thailand. Interventions that encourage retention in care and reinforce treatment adherence could further enhance the success of same‐day and rapid ART initiation.

## COMPETING INTERESTS

ST was funded as a grantee (Grant No. RGNS 65 – 041) from the Office of the Permanent Secretary, Ministry of Higher Education, Science, Research and Innovation, Thailand from 2022 to 2024.

## AUTHORS’ CONTRIBUTIONS

ST and NP created the study concept and study design. ST was responsible for data collection. NP oversaw programme implementation. ST conducted the analysis. NP and DCB advised on the analysis. ST drafted the manuscript. All authors critically reviewed the manuscript and approved the manuscript for submission.

## FUNDING

This work (Grant No. RGNS 65 – 041) was financially supported by the Office of the Permanent Secretary, Ministry of Higher Education, Science, Research and Innovation, Thailand from 2022 to 2024. This study was also supported by a Visiting Research Scholar grant and International SciKU Branding (ISB) funding from the Faculty of Science, Kasetsart University.

## DISCLAIMER

The content of this publication is solely the responsibility of the authors and does not necessarily represent the official views of any of the governments or institutions mentioned above.

## Supporting information



Supporting Information

## Data Availability

The data that support the findings of this study are available from the corresponding author upon reasonable request. Data were presented as an oral presentation at the 12th IAS Conference on HIV Science 2023 in Brisbane, Australia on 24/07/2023‐27/07/2023.
