# Supplementary material for: The impact of same‐day and rapid ART initiation under the Universal Health Coverage programme on HIV outcomes in Thailand: a retrospective real‐life cohort study
Source: J Int AIDS Soc. 2025 Jan 2;28(1):e26406. doi: 10.1002/jia2.26406 (PMC11695198; doi:10.1002/jia2.26406)
Supplement: Supplementary file 1 — Supporting Information [file JIA2-28-e26406-s001.docx]

**Supplementary Table 1 Characteristics of people living with HIV at ART initiation by ART initiation group (n=210,957).**

|  | **≤ 7 days** | **8 days to1 month** | **>1-3 months** | **>3 months** | **Total** |
| --- | --- | --- | --- | --- | --- |
| **N (%)** | 52,994 (25) | 55,067 (26) | 51,813 (25) | 51,083 (24) | 210,957 (100) |
| **Sex, N (%)** |  |  |  |  |  |
| Male | 36,615 (69) | 38,607 (70) | 36,775 (71) | 35,790 (70) | 147,787 (70) |
| Female | 16,379 (31) | 16,460 (30) | 15,038 (29) | 15,293 (30) | 63,170 (30) |
| **Age (years)** |  |  |  |  |  |
| **Median (IQR) age at ART initiation (years)** | 31 (24-41) | 33 (25-43) | 35 (26-44) | 34 (27-43) | 33 (26-43) |
| 15-24 years | 13,441 (25) | 12,625 (23) | 9,998 (19) | 8,355 (16) | 44,419 (21) |
| 25-34 years | 17,794 (34) | 16,837 (31) | 15,539 (30) | 17,506 (34) | 67,676 (32) |
| 35-49 years | 16,322 (31) | 18,540 (34) | 19,399 (37) | 19,493 (38) | 73,754 (35) |
| ≥ 50 years | 5,437 (10) | 7,065 (12) | 6,877 (14) | 5,729 (12) | 25,108 (12) |
| **First regimen** |  |  |  |  |  |
| NNRTI based | 44,140 (83) | 48,979 (89) | 47,695 (92) | 47,116 (92) | 187,930 (89) |
| PI based | 2,605 (5) | 1,330 (2) | 1,420 (3) | 1,791 (4) | 7,146 (3) |
| DTG based | 6,249 (12) | 4,758 (9) | 2,698 (5) | 2,176 (4) | 15,881 (8) |
| **HIV stage** |  |  |  |  |  |
| Asymptomatic HIV | 32,671 (62) | 32,274 (59) | 27,191 (53) | 29,610 (58) | 121,746 (58) |
| Symptomatic HIV | 7,474 (14) | 10,372 (19) | 9,469 (18) | 7,444 (15) | 34,759 (16) |
| AIDS | 12,849 (24) | 12,421 (22) | 15,153 (29) | 14,029 (27) | 54,452 (26) |
| **Year of ART initiation** |  |  |  |  |  |
| 2014-2016 | 9,180 (17) | 12,443 (23) | 16,668 (32) | 19,141 (37) | 57,432 (27) |
| 2017-2020 | 27,799 (52) | 29,042 (53) | 26,750 (52) | 24,614 (48) | 108,205 (51) |
| 2021-2022 | 16,015 (30) | 13,582 (25) | 8,395 (16) | 7,328 (14) | 45,320 (21) |
| **Region** |  |  |  |  |  |
| Bangkok | 13,772 (26) | 8,017 (15) | 6,918 (13) | 8,732 (17) | 37,439 (18) |
| Central | 9,112 (17) | 9,118 (17) | 8,600 (17) | 8,451 (16) | 35,281 (17) |
| Eastern | 5,583 (11) | 6,232 (11) | 5,916 (10) | 5,816 (11) | 23,547 (10) |
| Northeastern | 11,207 (21) | 14,255 (26) | 12,995 (25) | 11,355 (22) | 49,812 (24) |
| Northern | 8,024 (15) | 9,357 (17) | 8,077 (16) | 7,656 (15) | 33,114 (16) |
| Southern | 4,330 (8) | 6,089 (11) | 6,468 (13) | 6,340 (13) | 23,227 (11) |
| Western | 966 (2) | 1,999 (3) | 2,839 (6) | 2,733 (6) | 8,537 (4) |
| **CD4 at HIV diagnosis, N (%)** | 44016 (70) | 47517 (78) | 43973 (75) | 30231 (43) | 165737 (66) |
| **Median CD4 at HIV diagnosis** | 265 (105-433) | 187 (52-365) | 124 (36-314) | 289 (82-485) | 208 (58-398) |
| **Pre-ART CD4, N (%)** | 47,894 (90) | 51,771 (94) | 49,101 (95) | 45,217 (89) | 193,983 (92) |
| **Median Pre-ART CD4** | 307 (149-486) | 204 (59-385) | 146 (42-339) | 252 (96-441) | 229 (73-417) |
| < 200 | 15,464 (29) | 25,562 (47) | 28,488 (55) | 19,045 (37) | 88,559 (42) |
| 200-<350 | 11,695 (22) | 11,042 (20) | 8,858 (17) | 9,914 (19) | 41,509 (20) |
| 350-<500 | 9,449 (18) | 7,761 (14) | 6,001 (12) | 7,547 (15) | 30,758 (15) |
| ≥ 500 | 11,286 (21) | 7,406 (13) | 5,754 (11) | 8,711 (17) | 33,157 (16) |
| unknown | 5,100 (10) | 3,296 (6) | 2,712 (5) | 5,866 (12) | 16,974 (7) |

Abbreviations: ART, antiretroviral therapy; NNRTI, non-nucleoside reverse transcriptase inhibitor; PI, protease inhibitor; DTG, Dolutegravir; VL, viral load

Presented as n (%) for categorical data and median (interquartile range) for continuous data. The comparisons were performed using Pearson’s Chi-square tests or Fisher’s exact test, as appropriate, for categorical data, and Kruskal-Wallis tests for continuous data. All characteristics were statistically significant differences among ART initiation groups at 0.05.

Note: We included only youth living with HIV (84%, n=210,957/252,239) who had the ascertained date of HIV diagnosis to classify ART initiation group for sensitivity analysis.

**Supplementary Table 2 Factors associated with virological failure (plasma HIV RNA ≥ 1,000 copies/mL) after ART initiation (n=**210,957**).**

**Table 2 Factors associated with virological failure after ART initiation**

|  | **VF,**  **N (%)** | **Univariate** | | **Multivariate** | |
| --- | --- | --- | --- | --- | --- |
|  |  | **SHR (95%CI)** | **P** | **aSHR (95%CI)** | **P** |
| **ART initiation group** |  |  | <0.001 |  | <0.001 |
| ≤ 7 days | 3,927 (7) | reference (1) |  | 0.77 (0.73-0.80) |  |
| 8 days to 1 month | 3,789 (7) | 0.86 (0.82-0.90) |  | 1.10 (1.05-1.14) |  |
| >1-3 months | 5,798 (11) | 1.29 (1.24-1.35) |  | 1.48 (1.43-1.55) |  |
| >3 months | 6,859 (13) | 1.51 (1.45-1.57) |  | reference (1) |  |
| **Sex** |  |  | 0.003 |  | <0.001 |
| Male | 13,891 (9) | reference (1) |  | reference (1) |  |
| Female | 6,482 (10) | 1.05 (1.02-1.08) |  | 1.09 (1.06-1.12) |  |
| **Age at ART initiation (years)** |  |  | <0.001 |  | <0.001 |
| 15-24 years | 5,242 (12) | 2.04 (1.93-2.16) |  | 2.62 (2.47-2.79) |  |
| 25-34 years | 7,229 (11) | 1.76 (1.67-1.86) |  | 2.06 (1.94-2.18) |  |
| 35-49 years | 6,380 (9) | 1.36 (1.28-1.43) |  | 1.40 (1.32-1.48) |  |
| ≥ 50 years | 1,522 (6) | reference (1) |  | reference (1) |  |
| **First regimen** |  |  | <0.001 |  | <0.001 |
| NNRTI based ART | 18,884 (10) | 2.60 (2.29-2.95) |  | 1.16 (1.02-1.31) |  |
| PI based ART | 1,246 (17) | 4.28 (3.73-4.92) |  | 1.53 (1.33-1.75) |  |
| DTG based ART | 243 (2) | reference (1) |  | reference (1) |  |
| **HIV stage** |  |  | <0.001 |  | 0.74 |
| Asymptomatic HIV | 10,650 (9) | reference (1) |  | reference (1) |  |
| Symptomatic HIV | 3,480 (10) | 1.10 (1.06-1.14) |  | 0.99 (0.95-1.03) |  |
| AIDS | 6,243 (11) | 1.23 (1.19-1.27) |  | 1.01 (0.97-1.04) |  |
| **Year at ART initiation** |  |  | <0.001 |  | <0.001 |
| 2014-2016 | 8,643 (15) | reference (1) |  | reference (1) |  |
| 2017-2019 | 10,220 (9) | 0.73 (0.70-0.76) |  | 0.74 (0.71-0.78) |  |
| 2020-2022 | 1,510 (3) | 0.07 (0.07-0.08) |  | 0.08 (0.07-0.08) |  |
| **Pre-ART CD4, cells/mm^3^** |  |  | <0.001 |  | <0.001 |
| <200 | 11,552 (13) | 2.21 (2.10-2.31) |  | 2.18 (2.08-2.30) |  |
| 200-<350 | 3,463 (8) | 1.36 (1.28-1.43) |  | 1.43 (1.36-1.51) |  |
| 350-<500 | 2,063 (7) | 1.09 (1.03-1.16) |  | 1.12 (1.06-1.19) |  |
| ≥500 | 2,029 (6) | reference (1) |  | reference (1) |  |
| unknown | 1,266 (7) | 1.28 (1.19-1.37) |  | 0.77 (0.72-0.83) |  |
| **Region** |  |  | <0.001 |  | <0.001 |
| Bangkok | 3,167 (8) | reference (1) |  | reference (1) |  |
| Central | 3,568 (10) | 1.18 (1.12-1.23) |  | 0.96 (0.91-1.01) |  |
| Eastern | 2,448 (10) | 1.23 (1.17-1.30) |  | 1.00 (0.95-1.06) |  |
| Northeastern | 4,650 (9) | 1.10 (1.05-1.15) |  | 0.84 (0.80-0.88) |  |
| Northern | 3,203 (10) | 1.09 (1.04-1.15) |  | 0.90 (0.86-0.95) |  |
| Southern | 2,467 (11) | 1.20 (1.14-1.26) |  | 0.92 (0.87-0.97) |  |
| Western | 870 (10) | 1.16 (1.08-1.25) |  | 0.87 (0.80-0.94) |  |

SHR- sub-distribution hazard ratio; aSHR-adjusted sub-distribution hazard ratio, 95%CI-95%confidence interval

Abbreviations: ART, antiretroviral therapy; NNRTI, non-nucleoside reverse transcriptase inhibitor; PI, protease inhibitor, DTG- Dolutegravir, VF-virological failure.

N (%) was divided by a total of row.

Note: We included only youth living with HIV (84%, n=210,957/252,239) who had the ascertained date of HIV diagnosis to classify ART initiation group for sensitivity analysis.

**Supplementary Table 3 Factors associated with mortality and lost to follow-up after ART initiation (n=210,957).**

| **Covariates** | **Died, N (%)** | **Mortality** | | | | **LTFU, N (%)** | **Lost to follow-up** | | | |
| --- | --- | --- | --- | --- | --- | --- | --- | --- | --- | --- |
|  |  | **Univariable** | | **Multivariable** | |  | **Univariable** | | **Multivariable** | |
|  |  | **HR (95%CI)** | **P** | **aHR (95%CI)** | **P** |  | **HR (95%CI)** | **P** | **aHR (95%CI)** | **P** |
| **ART initiation group** |  |  |  |  |  |  |  |  |  |  |
| ≤ 7 days | 2,890 (5) | reference | <0.001 | reference | <0.001 | 6,822 (13) | reference | <0.001 | reference | <0.001 |
| 8 days to 1month | 4,786 (9) | 1.56 (1.48-1.63) |  | 1.23 (1.18-1.29) |  | 6,178 (11) | 1.67 (1.60-1.73) |  | 1.13 (1.09-1.17) |  |
| >1 to 3 months | 6,212 (12) | 2.02 (1.93-2.11) |  | 1.40 (1.34-1.47) |  | 3,946 (8) | 1.43 (1.38-1.49) |  | 1.06 (1.02-1.11) |  |
| >3 months | 6,497 (13) | 2.11 (2.02-2.20) |  | 1.67 (1.60-1.75) |  | 4,108 (8) | 0.95 (0.91-0.99) |  | 1.12 (1.07-1.16) |  |
| **Sex** |  |  |  |  |  |  |  |  |  |  |
| Male | 14,489 (10) | 1.08 (1.05-1.11) | <0.001 | 1.21 (1.17-1.24) | <0.001 | 15,191 (10) | 1.12 (1.08-1.15) | <0.001 | 0.98 (0.95-1.01) | 0.23 |
| Female | 5,896 (9) | reference |  | reference |  | 5,863 (9) | reference |  | reference |  |
| **Age at ART initiation (years)** |  |  |  |  |  |  |  |  |  |  |
| 15-24 years | 2,002 (5) | reference | <0.001 | reference | <0.001 | 5,758 (13) | reference | <0.001 | reference | <0.001 |
| 25-34 years | 4,938 (7) | 1.59 (1.51-1.67) |  | 1.30 (1.23-1.37) |  | 6,776 (10) | 0.77 (0.74-0.79) |  | 0.79 (0.76-0.82) |  |
| 35-49 years | 8,994 (12) | 2.66 (2.53-2.79) |  | 1.94 (1.85-2.04) |  | 6,162 (8) | 0.64 (0.62-0.67) |  | 0.73 (0.71-0.76) |  |
| ≥ 50 years | 4,451 (18) | 4.15 (3.94-4.37) |  | 3.1 (2.94-3.27) |  | 2,358 (9) | 0.75 (0.71-0.79) |  | 0.71 (0.67-0.74) |  |
| **First regimen** |  |  |  |  |  |  |  |  |  |  |
| NNRTI based | 19,228 (10) | 1.63 (1.49-1.77) | <0.001 | 1.62 (1.48-1.78) | <0.001 | 10650 (6) | 0.04 (0.04-0.04) | <0.001 | 0.10 (0.09-0.10) | <0.001 |
| PI based | 612 (9) | 1.28 (1.14-1.44) |  | 1.70 (1.51-1.92) |  | 447 (6) | 0.04 (0.04-0.05) |  | 0.12 (0.11-0.14) |  |
| DTG based | 545 (3) | reference |  | reference |  | 9957 (63) | reference |  | reference |  |
| **HIV stage** |  |  |  |  |  |  |  |  |  |  |
| Asymptomatic HIV | 8,888 (7) | reference | <0.001 | reference | <0.001 | 13,783 (11) | reference | <0.001 | reference | <0.001 |
| Symptomatic HIV | 4,253 (12) | 1.66 (1.60-1.72) |  | 1.27 (1.23-1.32) |  | 3,146 (9) | 0.80 (0.77-0.83) |  | 0.94 (0.90-0.98) |  |
| AIDS | 7,244 (13) | 1.78 (1.72-1.83) |  | 1.38 (1.34-1.43) |  | 4,125 (8) | 0.66 (0.64-0.69) |  | 0.85 (0.82-0.88) |  |
| **Year at ART initiation** |  |  |  |  |  |  |  |  |  |  |
| 2014-2016 | 8,083 (14) | reference | <0.001 | reference | <0.001 | 1283 (2) | reference | <0.001 | reference | <0.001 |
| 2017-2019 | 9,883 (9) | 0.81 (0.79-0.84) |  | 0.89 (0.86-0.92) |  | 4499 (4) | 1.87 (1.75-1.99) |  | 1.82 (1.70-1.94) |  |
| 2020-2022 | 2,419 (5) | 0.77 (0.74-0.80) |  | 0.89 (0.85-0.92) |  | 15272 (34) | 15.09 (14.22-16.01) |  | 7.37 (6.92-7.84) |  |
| **Pre-ART CD4, cells/mm^3^** |  |  |  |  |  |  |  |  |  |  |
| <200 | 13,032 (15) | 5.65 (5.29-6.05) | <0.001 | 4.14 (3.86-4.43) | <0.001 | 6,719 (8) | 0.93 (0.89-0.97) | <0.001 | 0.96 (0.91-1.00) | <0.001 |
| 200-<350 | 2,476 (6) | 2.18 (2.02-2.35) |  | 1.87 (1.73-2.02) |  | 3,492 (8) | 1.00 (0.95-1.05) |  | 1.01 (0.96-1.06) |  |
| 350-<500 | 1,106 (4) | 1.31 (1.20-1.43) |  | 1.24 (1.13-1.35) |  | 2,722 (9) | 1.05 (1.00-1.10) |  | 1.05 (1.01-1.10) |  |
| ≥500 | 910 (3) | reference |  | reference |  | 2,811 (8) | reference |  | reference |  |
| unknown | 2,861 (17) | 7.81 (7.25-8.41) |  | 7.18 (6.66-7.74) |  | 5,310 (31) | 4.66 (4.45-4.88) |  | 3.27 (3.12-3.43) |  |
| **Region** |  |  |  |  |  |  |  |  |  |  |
| Bangkok | 2,221 (6) | reference | <0.001 | reference | <0.001 | 3,636 (10) | reference | <0.001 | reference | <0.001 |
| Central | 3,551 (10) | 1.70 (1.61-1.80) |  | 1.44 (1.37-1.52) |  | 3,779 (11) | 1.11 (1.06-1.16) |  | 1.27 (1.22-1.33) |  |
| Eastern | 2,170 (9) | 1.56 (1.47-1.65) |  | 1.36 (1.28-1.44) |  | 2,433 (10) | 1.07 (1.02-1.13) |  | 1.37 (1.30-1.44) |  |
| Northeastern | 5,262 (11) | 1.81 (1.72-1.90) |  | 1.56 (1.48-1.64) |  | 4,923 (10) | 1.03 (0.99-1.08) |  | 1.22 (1.17-1.27) |  |
| Northern | 3,352 (10) | 1.69 (1.60-1.79) |  | 1.45 (1.37-1.53) |  | 3,234 (10) | 1.01 (0.96-1.05) |  | 1.25 (1.20-1.32) |  |
| Southern | 2,838 (12) | 2.05 (1.94-2.17) |  | 1.62 (1.53-1.71) |  | 2,195 (9) | 0.98 (0.93-1.03) |  | 1.27 (1.21-1.34) |  |
| Western | 991 (12) | 1.97 (1.83-2.12) |  | 1.53 (1.41-1.65) |  | 854 (10) | 1.04 (0.97-1.12) |  | 1.28 (1.19-1.38) |  |

HR- Hazard ratio; aHR-adjusted hazard ratio, 95%CI-95%confidence interval

N (%) was divided by a total of row.

Abbreviations: ART, antiretroviral therapy; NNRTI, non-nucleoside reverse transcriptase inhibitor; PI, protease inhibitor, DTG- Dolutegravir
